# 

**Published:** 1857-08

**Authors:** 


### CONTENTS, No. 2, Vol. IV.

ORIGINAL COMMUNICATIONS. Page

Poisoning by Corrosive Chloride of Mercury, - - 81
Death caused by Amylene, - - - 85
Reports of Case, by Prof. D. L. M'Gugin, M. D. - 88
American System of Medical Education. - - - - 95
Report of Cases, - - - - 103
Surgical Cases. Prof. J. C. Hughes. - - - - 110

DEPARTMENT OF SELECTIONS.

Treatment of Scarlatina by Diluted Acetic Acid, - - 115
Eunuchs, Circumcision and Leprosy in the East, - - 127
Artificial Respiration, - - - - - - - 132
Valvular Nature of Strangulated Hernia, - - 135
Incised wound of the Abdomen, - - - - 136
A New Poison, - - - - - - - 137

Progress of Medical Science. - - - - 138

EDITORIAL DEPARTMENT.

Meeting of the State Medical Society, - - - 149
"Independent"—Gunn, - - - - - 153
Medical Appointment—Prof. Armor, - - 154
" " Prof. Rauch, 155
Notice of Advertisements, - - - - 156
Prof. N. S. Davis' Letter, - - - - 157
Free Medical University, - - - - - - 158
Errata &c. in our last Number, - - - - - 159
Miscellaneous Items and News, - - - - - 159

